# IMA Genome-F 11: Draft genome sequences of *Fusarium xylarioides, Teratosphaeria gauchensis* and *T. zuluensis* and genome annotation for *Ceratocystis fimbriata*

**DOI:** 10.1186/s43008-019-0013-7

**Published:** 2019-09-13

**Authors:** Brenda D. Wingfield, Arista Fourie, Melissa C. Simpson, Vuyiswa S. Bushula-Njah, Janneke Aylward, Irene Barnes, Martin P. A. Coetzee, Léanne L. Dreyer, Tuan A. Duong, David M. Geiser, Francois Roets, E. T. Steenkamp, Magriet A. van der Nest, Carel J. van Heerden, Michael J. Wingfield

**Affiliations:** 1 Department of Biochemistry, Genetics and Microbiology (BGM), Forestry and Agricultural Biotechnology Institute (FABI), University of Pretoria, Private Bag X20, Hatfield, 0028 South Africa; 2 Department of Botany and Zoology, Stellenbosch University, Private Bag X1, Matieland, 7602 South Africa; 3 Fusarium Research Center, Department of Plant Pathology and Environmental Microbiology, 121 Buckhout Lab, University Park, State College, PA 16802 USA; 4 Department of Conservation Ecology and Entomology, Stellenbosch University, Private Bag X1, Matieland, 7602 South Africa; 5 Central Analytical Facilities, Stellenbosch University, Private Bag X1, Matieland, 7602 South Africa; 6 Biotechnology Platform, Agricultural Research Council, Private Bag X05, Onderstepoort, 0002 South Africa

**Keywords:** Genome announcement, Genome annotation, Fungal pathogen, Coffee pathogen, Eucalyptus canker pathogen, *Fusarium xylarioides*, *Teratosphaeria gauchensis*, *T. zuluensis*

## Abstract

Draft genomes of the fungal species *Fusarium xylarioides, Teratosphaeria gauchensis* and *T. zuluensis* are presented. In addition an annotation of the genome of *Ceratocystis fimbriata* is presented. Overall these genomes provide a valuable resource for understanding the molecular processes underlying pathogenicity and potential management strategies of these economically important fungi.

## IMA GENOME-F 11A

### Genome annotation for *Ceratocystis fimbriata*: an aggressive fungal pathogen of root crops

#### Introduction

The genus *Ceratocystis* includes 41 species of mainly plant pathogenic fungi (Marin-Felix et al. 2017; Holland et al. 2019 Barnes et al. 2018; Liu et al. 2018). The type species, *Ceratocystis fimbriata*, was first described in the USA in 1890 as the causal agent of black rot of *Ipomoea batatas* (Halsted 1890). It shows strong host specificity and does not infect tree hosts, in contrast to many other species in this genus (Baker et al. 2003; Fourie et al. 2018). The pathogen is known in various parts of the USA as well as in Asian countries including Papua New Guinea, China, Japan, Hawaii and New Zealand (Halsted and Fairchild 1891; Lewthwaite et al. 2011; Li et al. 2016). Population genetic analyses of isolates collected in these countries have revealed a very low genetic diversity in all the populations and *C. fimbriata* thus appears to be a near clonal species (Li et al. 2016; Scruggs et al. 2017).

The sexual fruiting structures in *Ceratocystis* spp., including *C. fimbriata*, are flask-shaped ascomata with long necks exuding sticky ascospore masses at their apices (Wingfield et al. 2017b). *Ceratocystis fimbriata*, along with all other species of *Ceratocystis*, is self-fertile (Halsted 1890; De Beer et al. 2014). The fungus includes isolates that are self-sterile, arising from a phenomenon known as unidirectional mating-type switching, where an isolate of the opposite mating-type is required for sexual reproduction to occur (Harrington and McNew 1997; Witthuhn et al. 2000; Wilken et al. 2014).

Genome sequences represent valuable scientific resources that provide an important source of information needed to understand the biology of organisms. A draft genome sequence of *C. fimbriata* (isolate CBS114723) was published in 2013 (Wilken et al. 2013) and these data were specifically used to characterise the mating-type locus of the isolate (Wilken et al. 2014). Genomes of other species of *Ceratocystis*, including *C. albifundus* (Van der Nest et al. 2014a)*, C. eucalypticola* (Wingfield et al. 2015b) and *C. manginecans* (Van der Nest et al. 2014b), have also been published, and form part of a larger genome sequencing project. The aim of this study was firstly to improve the genome assembly of *C. fimbriata*, the type species of this genus, and secondly to provide a curated annotation of this genome that can serve as a resource for other species in the genus. Here, we present an improved assembly of *C. fimbriata* with significantly fewer contigs than the previous assembly, and the first annotation of this genome http://www.ncbi.nlm.nih.gov/Taxonomy/Browser/wwwtax.cgi?lvl=0&id=5158*,* with RNA data incorporated to improve gene prediction. Genomes of Ceratocystidaceae provide an opportunity to investigate various taxonomic and evolutionary questions of an important group of plant pathogens.

#### Sequenced strains

USA: North Carolina, isolated from *Ipomoea batatas,* Dec, 1998, D. McNew (CMW14799, C1421, CBS114723).

#### Nucleotide sequence accession number

The genomic sequence of *Ceratocystis fimbriata* (CMW14799, CBS114723) has been deposited at DDBJ/EMBL/GenBank under accession no. APWK00000000. The version described in this paper is version APWK03000000. RNA sequencing data has been deposited in the NBCI Short Read Archive under accession number PRJNA67151.

#### Materials and methods

*Ceratocystis fimbriata* was grown on 2% (w/v) MEA (Biolab, Merck, South Africa), supplemented with 150 mg/L streptomycin and 100 μg/L thymine (MEA-ST medium), at room temperature (22–24 °C) for two weeks. DNA extraction was performed using the protocol described by Goodwin et al. (1992) with minor modifications. An RNase treatment step was included after the first phenol/chloroform step and incubated for 1 h at 37 °C. The RNase was then removed with a subsequent phenol/chloroform step. DNA purity was determined using a ND_1000 Spectrophotometer (Nanodrop, Wilmington, DE). The integrity of the genomic DNA was determined with agarose gel electrophoresis on a 1% gel. DNA quantity was determined with a Qubit® 2.0 Fluorometer (ThermoFisher Scientific, Waltham, USA) following the manufacturer’s protocol.

To verify the identity of the isolate and the absence of bacterial contamination, the fungal ITS regions 1 and 2, including the 5.8S rRNA gene (refered collectively as the ITS region) of the ribosomal operon and a portion of the bacterial 16S rRNA were amplified. Amplification and amplicon purification of the ITS region were performed, as described by Fourie et al. (2015), using primers ITS1 and ITS4 (White et al. 1990). The sequence of the PCR product was determined with Sanger sequencing using an ABI Big DYE Terminator Cycle Sequencing Ready Reaction Kit (Applied BioSystems, Thermo Fisher, California, USA). The ITS sequence was then aligned against that of *C. fimbriata*. The 16S rRNA region was amplified using primers 27F and 1492R (DeLong 1992) and PCR cycler conditions from Beukes et al. (2013), including a positive control bacterial DNA sample. The absence of a PCR band confirmed the absence of bacterial DNA in the sample.

The taxonomic placement of *C. fimbriata* among the Microascales was investigated by means of a phylogenetic analysis of three combined gene regions, the 28S and 60S ribosomal RNA and the Mcm7 (DNA replication licensing factor) gene regions. The sequences of representative isolates of the different genera in this order were obtained from GenBank, as reported by De Beer et al. (2014), and aligned using Muscle alignment in MEGA v.7 (Kumar et al. 2016). A maximum likelihood analysis was performed with the sequence data, using RaxML v.8.2.11 (Stamatakis 2014), model parameters were estimated by the software and 1000 bootstrap replicates were performed to obtain branch support values. The *Graphium* genus was selected as outgroup.

Genome sequencing was performed by FASTERIS SA, Switzerland. All libraries were combined in one lane of an Illumina HiSeq 2500 instrument that produces reads of 125 bp in length. Three libraries were constructed; consisting of a 3000 bp insert mate-pair library and both a 300 bp and 500 bp paired-end library. Illumina adapter sequences were removed from the reads by FASTERIS. Initial quality control (QC) of the sequence reads of each library was performed using FastQC (Andrews 2010). Low quality reads (leading and trailing Phred score < 20) and reads shorter than 20 bp (mate-pair) and 100 bp (paired-end), respectively, were removed from the data using Trimmomatic (Bolger et al. 2014). Analysis with FastQC was repeated, after read trimming, to ensure all reads had a Phred quality score above 20 and that all adapters were removed.

Genome assemblies were performed using Velvet Optimiser v.2.2.5 (Zerbino and Birney 2008) optimising for k-mer values between 61 and 99. Velvet Optimiser indicated a k-mer value of 99 to be optimal. All contigs below 500 bp were discarded. The assembled contigs were subsequently joined into scaffolds by incorporating the mate-pair library data, using the program SSPACE v.2 (Boetzer et al. 2011). Parameters were set to extend contigs, using unmapped reads and five read pairs were required to support the joining of two contigs for the creation of scaffolds. Raw reads were mapped back to the genome to fill in gaps within scaffolds using the software GapFiller v.1.10 (Boetzer and Pirovano 2012). Standard parameters were retained except that a minimum overlap of 100 bp was selected for reads mapped back to the scaffolds and read trimming was switched off. The pipeline BUSCO (Benchmarking Universal Single-Copy Orthologs; Simão et al. 2015) was used to determine the percentage of conserved Ascomycete (1315 genes) and Sordariomycete (3375 genes) single-copy orthologs present in the genome.

RNA extraction was performed on mycelial and ascomatal tissue harvested from three agar plates after ten days of growth. Liquid nitrogen was used to flash freeze the harvested tissue after which a mortar and pestle was used to grind it to a fine powder. RNA extractions were then performed using the RNeasy® Plant Mini Kit (Qiagen, Limburg, The Netherlands) following the manufacturer’s instructions but the RLC buffer was replaced with RLT buffer, and the optional DNase-1 digestion step was included. Quality of the extracted total RNA was evaluated using agarose gel electrophoresis with 2% (w/v) agarose (Seakem). The concentration was then measured using a ND_1000 Spectrophotometer (Nanodrop, Wilmington, DE). Further quality assessment was performed using the Experion™ automated electrophoresis system (Bio-Rad Laboratories, California, USA). The extracted RNA was enriched for mRNA using Dynabead® mRNA purification kit (ThermoFisher Scientific, Waltham, USA) and then subjected to cDNA synthesis, library preparation and sequencing at the Central Analytical Facilities, Stellenbosch University, South Africa, using the Ion Proton Platform and PI™ Chip system (Life Technologies, Carlsbad, CA). Quality of the raw reads was checked using FastQC as described above. Sequences were trimmed using Trimmomatic by removing low quality reads (leading and trailing Phred score < 20), reads below 20 bp in length, the first 20 bp of each read (due to low quality), and any bases over 300 bp in length.

The MAKER genome annotation pipeline v2.31.8 (Cantarel et al. 2008; Holt and Yandell 2011) was used for structural annotation of the genome. RNA-Seq data was incorporated into training of gene predictors and used as gene evidence during all MAKER iterations. The RNA-Seq reads were aligned to the genome with STAR (Dobin et al. 2013) and transcripts were assembled with Cufflinks (Trapnell et al. 2012). The aligned RNA-Seq reads in BAM format were incorporated into the Braker1 pipeline (Hoff et al. 2016) for training of AUGUSTUS v.3.2.1 (Stanke et al. 2004) and GeneMark-ET (Lomsadze et al. 2014). SNAP (Korf 2004) was trained with transcripts assembled from Cufflinks using the est2genome function in MAKER. A species specific repeat library was created using RepeatScout (Price et al. 2005) and RepeatMasker (Smit et al. 1996–2010). This repeat library was used to mask the genome against repetitive elements before performing gene prediction in MAKER. A final MAKER run was conducted using trained parameters from SNAP, AUGUSTUS and GeneMark. FgeneSH (Solovyev et al. 2006) was run based on parameters pre-optimised for *Neurospora crassa* and the gene models obtained were passed over to MAKER in the final run. Gene models obtained from MAKER were visualised using WebApollo (Lee et al. 2013), along with RNA-seq evidence aligned to the genome, and all gene models were examined and curated, where necessary, based on the RNA-Seq evidence.

Functional annotation of the genes was based on comparison to various databases. A BLASTp analysis was performed for all proteins against the Swissprot database. Proteins were also compared to the Protein family (Pfam) database using InterProScan v.5.24 (Jones et al. 2014). SignalP v.4.0 (Petersen et al. 2011) was used to predict secretion signals and Phobius v.1 (Käll et al. 2004) was used to predict the presence of transmembrane domains. To predict the number of genes present in internal clusters, the genes involved in secondary metabolite production were determined using the antiSMASH v.3 software available online (Weber et al. 2015). The functional annotations predicted for each protein were added to the gff file using the software ANNIE (Ooi et al. 2009) and GAG (Hall et al. 2014).

#### Results and discussion

**Fig. 1:**
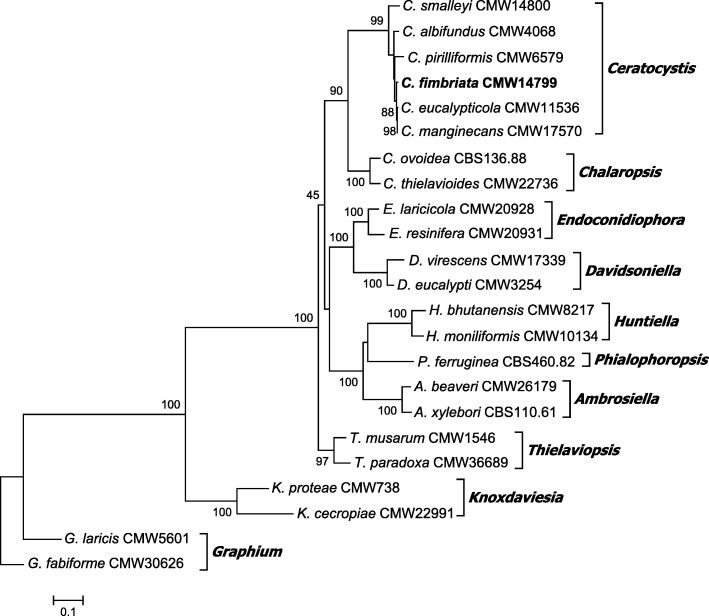
Phylogenetic tree depicting the relationship between *C. fimbriata* (in bold) and related species and genera in the Microascales. The tree was constructed from 60S, LSU, MCM7 gene regions using RaxML. Bootstrap support (1000 replicates) are indicated at the nodes

The paired-end and mate-pair sequencing generated approximately 42,5 million raw reads with an average length of 166 bp. Trimmed reads were assembled into 399 scaffolds, ranging in size from 500 bp to 516 595 bp, with an average read coverage of 630 and N50 value of 173 733 bp. The *C. fimbriata* genome was 30 159 98 bp in size with a GC content of 45.6%. There were 7728 predicted genes, of which 7266 (94%) were protein-coding genes, 105 (1.36%) were rRNA genes and 348 (4.5%) were tRNA genes. In total, 62% of the genes could be annotated with a known function. The *C. fimbriata* genome contained 98% Ascomycete and 90% Sordariomycete completed BUSCO gene models. The taxonomic placement of *C. fimbriata* among the Microascales is illustrated in Fig. 1. The sequence alignments were submitted to Treebase (24031).

This study showed that *C. fimbriata* has far fewer genes than other fungal plant pathogens with similar genome sizes. For example, the head blight pathogen *Fusarium graminearum* (genome size: 36.5 Mb) has approximately 14 164 genes (King et al. 2015), the pine needle blight pathogen *Dothistroma septosporum* (genome size: 31.2 Mb) has 12 580 genes (De Wit et al. 2012) and the wilt pathogen *Verticillium dahliae* (genome size: 33.8 Mb) has 10 535 genes (Klosterman et al. 2011). The number of genes in *C. fimbriata* does, however, correspond with those of other *Ceratocystis* species (Van der Nest et al. 2014a; Van der Nest et al. 2014b; Wingfield et al. 2015b; Wingfield et al. 2016b). There are currently eight genomes publicly available for species of *Ceratocystis* and 18 in the Ceratocystidaceae (Wilken et al. 2013; Van der Nest et al. 2014a; Van der Nest et al. 2014b; Van der Nest et al. 2015; Wingfield et al. 2015a; Wingfield et al. 2015b; Wingfield et al. 2016a; Wingfield et al. 2017; Molano et al. 2018; Vanderpool et al. 2018; Wingfield et al. 2018a). The annotation of the *C. fimbriata* genome sequence presented in this study is the first manually curated genome including additional RNA evidence, for any species in the Ceratocystidaceae. This annotation will provide the means to improve annotations for other species of *Ceratocystis* and provides an improved assembly that can be used for future comparative studies.

*Authors:* A. Fourie*, M.C. Simpson*, T.A. Duong, I. Barnes, M.P.A. Coetzee, M.A. van der Nest, M.J. Wingfield and B.D. Wingfield.

*Contact: melissa.simpson@fabi.up.ac.za or arista.fourie@fabi.up.ac.za (authors contributed equally)

## IMA GENOME-F 11B

### Draft genome sequence of *Fusarium xylarioides*

#### Introduction

*Fusarium xylarioides* Steyaert (1948) is a soilborne fungal pathogen that causes coffee wilt disease (CWD) in many coffee growing regions in Africa (Rutherford 2006). The sexual stage, previously referred to as *Gibberella xylarioides* (Heim 1950), is readily observed in CWD infested trees (Ploetz 2006). This heterothallic fungus is a member of the *F. fujikuroi* species complex (FFSC), a group of phylogenetic species that infect a number of important crops (O’Donnell et al. 1998; O’Donnell et al. 2000). Previous studies have reported the presence of two genetically and biologically distinct forms of this pathogen (Geiser et al. 2005; Lepoint et al. 2005), but their taxonomy has not yet been conclusively resolved. The availability of a complete genome sequence will, therefore, serve as the starting point for addressing the taxonomic confusion about this species in the literature. Also, more genetic data on this species will enable studies on its biology and evolution.

#### Sequenced strain

Uganda: Iganga District: Isolated from *Coffea canephora*, Dec. 2000, *D.M. Geiser* (KSU 18978 = FRC L-0394 = CMW 53787 – living culture).

#### Nucleotide sequence accession number

The whole genome shotgun sequencing project of *Fusarium xylarioides* KSU 18978 (FRC-L0394 = CMW 53787) has been deposited at DDBJ/ENA/GenBank under the accession no. SRZU00000000. The version described in this paper is version SRZU01000000.

#### Materials and methods

*Fusarium xylarioides* KSU 18978 (CMW 53787) was obtained from the culture collection (CMW) of the Forestry and Agricultural Biotechnology Institute (FABI), University of Pretoria. The isolate was grown in 40 ml potato dextrose broth (20% potato dextrose broth w/v) and incubated on an orbital shaker (135 rpm)for 2 d at room temperature (22–25 °C). Genomic DNA was extracted following the method of Duong et al. (2013). One pair-end library of 250 bp read length and 550 bp insert size was prepared and sequenced using the Illumina HiSeq 2500 platform. Quality control of pair-end reads received and adapter trimming was performed in the program Trimmomatic v. 0.36 (Bolger et al. 2014). Genome assembly was performed from trimmed reads using SPAdes v. 3.10 (Bankevich et al. 2012) and further scaffolding was performed using SSPACE Standard v. 3.0 (Boetzer et al. 2011). The genome quality and completeness were evaluated with BUSCO v. 2.0 (Simão et al. 2015) using the dataset for *Sordariomycetes*. The number of protein-coding genes encoded by the genome was evaluated with the program AUGUSTUS v. 3.2.2 (Stanke et al. 2006) using *Fusarium graminearum* as a species model. The taxonomic identity of the sequenced genome was confirmed by Maximum likelihood (ML) analysis of authenticated sequences using MEGA version X (Kumar et al. 2018).

#### Results and discussion

The assembled draft genome of *Fusarium xylarioides* was estimated to be 55.24 Mb with a coverage of 61x, corresponding to 424 scaffolds larger than 500 bp with an N50 value of 250 204 bp, and an average GC content of 43.4%. Among the FFSC species for which genomes sequences are available, *F. xylarioides* thus has the largest predicted genome size, i.e., the genomes of *F. circinatum* is 43.43 Mb, *F. temperatum* 45.46 Mb, *F. fracticaudum* 46.29 Mb, *F. pininemorale* 47.83 Mb, and *F. nygamai* is 51.61 Mb (Wingfield et al. 2015a, 2015b, 2017, 2018a). The predicted size of our sequenced genome was similar to that of another *F. xylarioides* strain K1 (55.11 Mb), whose genome sequence has recently been made available on NCBI under nucleotide accession number GCA_004329255.

Based on BUSCO analysis, genome completeness was 99.0% (C:3687 [S:3680, D:7], F:26, M:12, n:3725), suggesting that the assembly covers the majority of the organism’s gene content. AUGUSTUS predicted that the assembly encodes 14 588 open reading frames (ORFs) and this was in agreement with data from other members of the FFSC (Wingfield et al. 2015a, 2015b, 2017, 2018a).

**Fig. 2:**
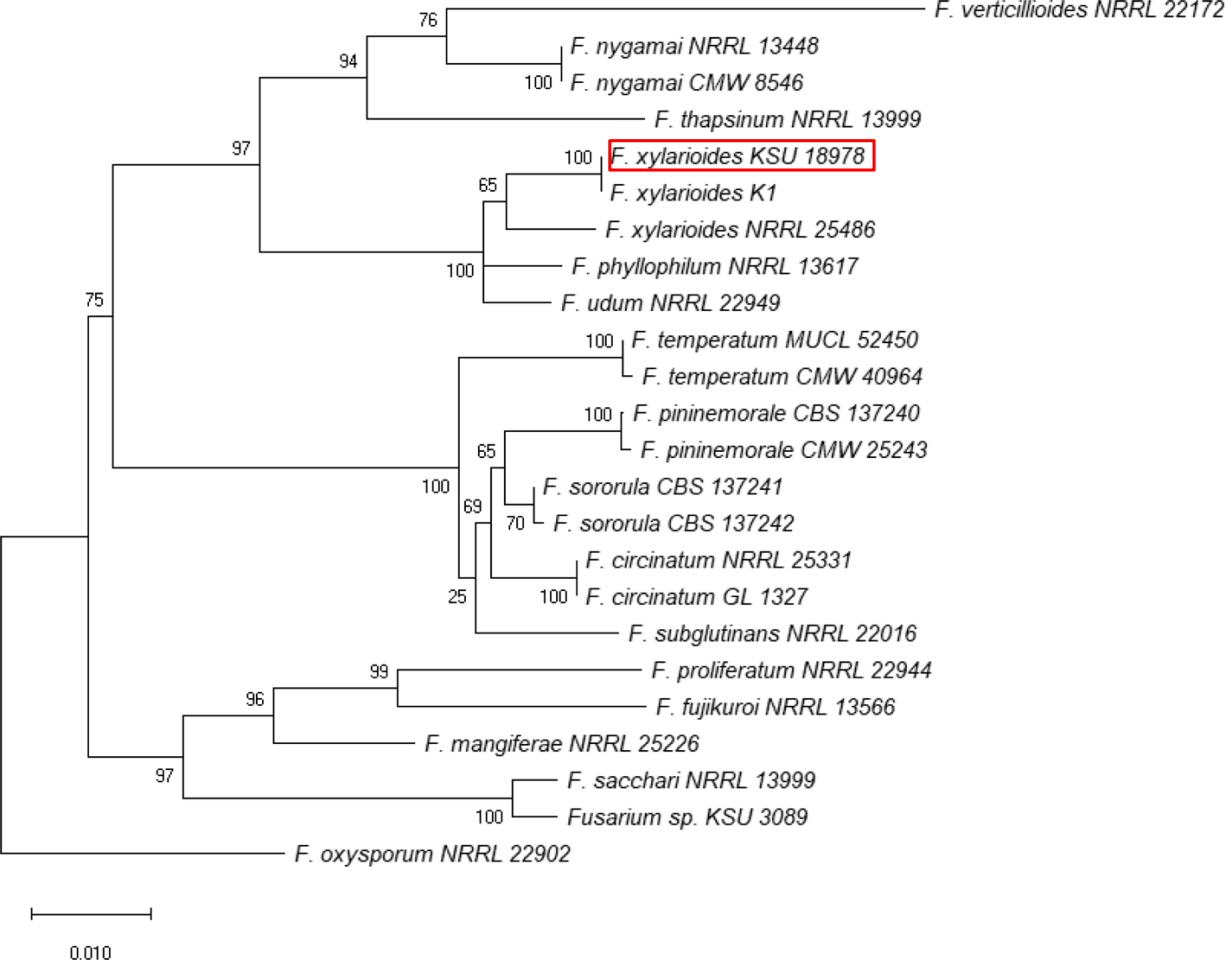
Maximum likelihood (ML) tree based on partial gene sequences of β-tubulin and translation elongation factor 1-α. Sequence alignments were assembled with MAFFT version 7 (Katoh and Standley 2013). The program MEGA X version 7 (Kumar et al. 2018) was used to estimate the best-fit substitution model (TN93) with a discrete gamma distribution (*+G*). The same program was used for ML phylogenetic analysis and percentage bootstrap support (1000 replications) values are indicated at branch nodes

Phylogenetic analysis based on partial gene sequences of β-tubulin and TEF-1α confirmed the sequenced genome to be of *F. xylarioides* (Fig. 2). *F. xylarioides* and *F. udum* are the only two members of the FFSC that cause true vascular wilt diseases (Geiser et al. 2005). Therefore, the increase in available whole genome data will not only allow for taxonomic re-evaluation of *F. xylarioides*, but will also enable comparative genomics studies to better understand the biology and evolution of members of the FFSC.

*Authors:* V.S. Bushula-Njah*, T.A. Duong, D.M. Geiser, E.T. Steenkamp, and B.D. Wingfield.

*Contact: Vuyiswa.bushula@fabi.up.ac.za

## IMA GENOME-F 11C

### Draft genome sequences of *Teratosphaeria gauchensis* and *T. zuluensis*, causal agents of Teratosphaeria stem canker

#### Introduction

Teratosphaeria stem canker (previously known as Coniothyrium canker) is a fungal disease of *Eucalyptus* trees planted outside of their native range for the production of wood and non-wood products. Since its discovery in a South African plantation in the late 1980’s (Wingfield et al. 1996), this disease has emerged in *Eucalyptus* plantations in 14 additional countries where tropical or subtropical climates predominate (Aylward et al. 2019). Infection is characterized by necrotic lesions that grow and eventually merge to form large gum-filled, bleeding cankers (Wingfield et al. 1996). Gum-stained wood is unsuitable for timber production and lesions hinder de-barking so that pulping is affected (Old et al. 2003).

**Fig. 3:**
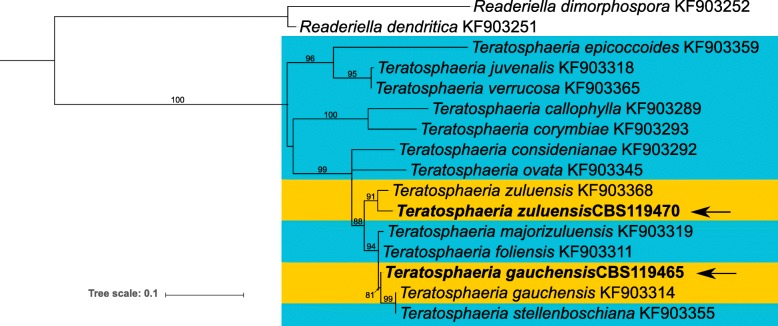
Maximum Likelihood phylogeny of the Teratosphaeria stem canker pathogens and related species based on the Elongation Factor (EF1-α) gene. Leaf-associated species are highlighted in teal, whereas the stem canker pathogens are yellow. The genome isolates described in this study are indicated with arrows. GeneBank accessions are shown after the species name

*Teratosphaeria gauchensis* and *T. zuluensis*, Dothideomycete fungi (Capnodiales, Teratosphaeriaceae), cause Teratosphaeria stem canker independently (Cortinas et al. 2006). These two species are unique in that they are the only known stem canker pathogens in a genus predominantly associated with the leaves of *Eucalyptus* species (Fig. 3; Quaedvlieg et al. 2014). Until recently, they were thought to have distinct geographic distributions, but in 2014 both were identified in a Ugandan plantation (Jimu et al. 2014). Together with the 2015 discovery of *T. gauchensis* in southern Africa (Jimu et al. 2015), this suggests that concurrent infections are bound to occur in plantations of central and southern Africa (Aylward et al. 2019).

We present the genome sequences of the ex-holotype of *T. gauchensis* (CBS 119465) and the ex-type of *T. zuluensis* (CBS 119470). These genomes will enable exploration of the intriguing case of two different species causing identical disease symptoms. Future studies will focus on the eucalypt stem specificity of these pathogens in contrast to the widespread eucalypt leaf association of other *Teratosphaeria* species.

#### Sequenced strain

*Teratosphaeria gauchensis*: Uruguay: *La Juanita*: isol. Stem cankers on *Eucalyptus grandis*, Feb. 2005, *M.J. Wingfield* (CBS 119465 = CMW 50181- culture, PREM 62331 – dried culture).

*Teratosphaeria zuluensis*: South Africa: *KwaZulu-Natal Province* (Kwambonambi plantation): isol. Stem cankers on *Eucalyptus grandis*, Feb. 2005, *M.J. Wingfield* (CBS 119470 = CMW 50183 – culture, PREM 62332 – dried culture).

#### Nucleotide accession number

The genomic sequence data of *T. gauchensis* and *T. zuluensis* have been deposited at DDJ/EMBL/GenBank under the accessions VCMR00000000 (*T. gauchensis*) and VCMQ00000000 (*T. zuluensis*). This paper describes the first versions of these genomes.

#### Material and methods

Fungi were cultured on 2% Malt Extract Agar (Merck, Wadeville, South Africa) at 25 °C for approximately 2 weeks. DNA was extracted as described for *T. destructans* (Wingfield et al. 2018b). The quality of the extracted DNA was estimated from the absorbance curve and the 260/280 and 260/230 absorbance ratios determined by a NanoDrop ND-1000 spectrophotometer (ThermoFisher Scientific, Wilmington, USA). DNA concentrations were determined with a Qubit® 2.0 Fluorometer (Invitrogen, Carlsbad, California).

*Teratosphaeria gauchensis* (CBS 119465) was sequenced at Macrogen (Seoul, Korea) using a single PacBio Sequel SMRT Cell as well as a portion of an Illumina HiSeq 2500 lane at Inqaba Biotec (Pretoria, South Africa). The single paired-end Illumina library had an insert size of 550 bp and a target read length of 250 bp. A hybrid assembly was computed by trimming the raw Illumina sequence reads with Trimmomatic 0.38 (Bolger et al. 2014) and using these in LoRDEC 0.6 (Salmela and Rivals 2014) to correct the PacBio reads. The corrected PacBio reads were assembled with Canu 1.7.1 (Koren et al. 2017). An Illumina assembly was subsequently constructed in SPAdes 3.10.1 (Bankevich et al. 2012), using k-mer values of 21, 33, 55, and 77 and applying the Canu assembly as “trusted reads”. A final error correction of the hybrid assembly was performed with Pilon 1.22 (Walker et al. 2014).

*Teratosphaeria zuluensis* (CBS 119470) was sequenced with the Ion 520™ & Ion 530™ ExT Kit and an Ion 530™ Chip (ThermoFisher Scientific, MA) at the Central Analytical Facility (CAF, Stellenbosch University, South Africa). Assembly of reads was done with SPAdes 3.10.1 (Bankevich et al. 2012) using k-mer values 21, 33, 55, 77, 99, and 127. For both *T. gauchensis* and *T. zuluensis*, genome completeness was estimated with Benchmarking Universal Single-Copy Orthologs (BUSCO) 2.0.1 (Simão et al. 2015) using the “Ascomycota *odb9*” dataset.

Repeats within each genome were identified with RepeatScout and masked with RepeatMasker Open-4.0.7 (http://www.repeatmasker.org). Annotation was performed with the MAKER 2.31.10 pipeline (Campbell et al. 2014) using pre-trained de novo gene predictors AUGUSTUS 3.3 (Stanke et al. 2006), GeneMark-ES Suite 4.35 (http://exon.gatech.edu/GeneMark/) and SNAP 2006-07-28 (Korf 2004). External EST and protein evidence from 15 other Capnodiales species were included in the annotation pipeline.

The Elongation Factor genes (EF1-α) of the two stem canker pathogens were extracted from their genomes and used along with the EF1-α genes of related species (Quaedvlieg et al. 2014) for phylogenetic analysis. The Maximum Likelihood tree was computed with the PhyML+SMS “one-click” method on NGPhylogeny.fr (Lemoine et al. 2019). This protocol employs MAFFT (Katoh et al. 2017) for multiple alignment, BMGE (Criscuolo and Gribaldo 2010) for alignment curation and the aLRT SH-like method (Anisimova and Gascuel 2006) for calculating bootstrap support.

#### Results and discussion

*Teratosphaeria gauchensis* was assembled into 53 contigs larger than 1 kb, with an N50 = 1.44 Mb and L50 = 8. This equated to a total assembly size of 30.27 Mb with a GC content of 45.6%. The PacBio reads provided an estimated genome coverage of 124x with an additional 160x coverage obtained from the Illumina data. The *T. zuluensis* assembly yielded 86 contigs above 1 kb (N50 = 1.00 Mb, L50 = 12), an estimated genome size of 28.71 Mb, 44.5% GC and estimated 155x genome coverage. The search for 1315 Ascomycota ortholog proteins identified 97.6% (1285) single-copy orthologs in *T. gauchensis* and 96.9% (1274) in *T. zuluensis*, indicating that these genomes are ca. 97% complete. This presents a higher level of completeness than the 84.5% of *T. destructans*, the only other published *Teratosphaeria* genome (Wingfield et al. 2018b).

A similar number of genes were predicted in *T. gauchensis* and *T. zuluensis* at 11699 and 11520, respectively. Of these, 9304 predictions in *T. gauchensis* and 9457 in *T. zuluensis* were supported by external evidence. Both genomes had a low repeat content of 2.2%, much lower than the ca.17% estimated for *T. destructans* (GenBank RIBY01000000). Since the genomes of these stem canker pathogen species, as well as *T. destructans*, were sequenced with long-read technologies, we are confident that this is not an underestimate, but reflects the true repeat content in these genomes.

The EF1-α gene tree (Fig. 3) illustrates the relationship of *T. zuluensis* and *T. gauchensis* to leaf-associated *Teratosphaeria* species. This phylogeny, as well as others (Aylward et al. 2019; Quaedvlieg et al. 2014), suggests that the association with *Eucalyptus* stem cankers evolved more than once. The *T. zuluensis* genome isolate CBS119470 groups with the ex-epitype strain CBS120301 (KF903368), however, the *T. gauchensis* strain sequenced in this study (CBS119465) groups sister to a clade that contains an ex-type strain (CBS120304; KF903314) of *T. gauchensis* and *T. stellenboschiana*. A similar phylogenetic relationship is apparent with both the ITS and β-tubulin gene regions as well as with concatenated gene trees (data not shown). *Teratosphaeria gauchensis* is known to represent a species complex displaying statistical support for subgroups within the species (Aylward et al. 2019; Silva et al. 2015). Future studies should take the taxonomic position of this genome isolate into consideration when interpreting genomic data.

Several other fungal groups affect *Eucalyptus* stems*,* including species in the genera *Chrysoporthe* (Gryzenhout et al. 2004), *Cytospora* (Adams et al. 2006), *Holocryphia* (Van der Westhuizen et al. 1993) and *Neofusicoccum* (Slippers et al. 2009). Of these, genomes of *Eucalyptus*-specific isolates are available only for *Chrysoporthe austroafricana*, *C. cubensis* and *C. deuterocubensis*, the causal agents of Cryphonectria canker (Wingfield et al. 2015a, 2015b). *Teratosphaeria gauchensis* and *T. zuluensis*, therefore, represent the second group of *Eucalyptus* stem canker pathogens to be sequenced. In future, comparative genomics projects that include various *Eucalyptus* stem pathogens as well as closely related species associated with other plant organs (e.g. *T. destructans*), may reveal fungal characteristics that enable stem pathogenicity.

*Authors:* **J. Aylward, B.D. Wingfield, L.L. Dreyer, F. Roets, C.J. van Heerden, M.J. Wingfield.**

*Contact*: Janneke.Aylward@fabi.up.ac.za

## Data Availability

All data and material is available the relevant details (data banks, culture collections and herbaria) are given in the manuscript.

## Abbreviations

aLRT SH: Aproximate Likelihood Ratio Test for branches
ANNIE: ANNotation Information Extractor
AUGUSTUS: Software for gene prediction
BLAST: Basic Local Alignment Search Tool
BLASTp: Basic Local Alignment Search Tool for protein sequences
BMGE: Block Mapping and Gathering with Entropy
BUSCO: Benchmarking Universal Single-Copy Orthologs
Canu: A hierarchical assemblypipeline
cDNA: Complementary Deoxyribose Nucleic Acid
CWD: Coffee wilt disease
DDBJ: DNA Data Bank of Japan
DNA: Deoxyribose Nucleic Acid
DST: Department of Science and Technology of South Africa
EF1-α: Elongation Factor 1 alpha gene
EMBL: Nucleotide Sequence Data Library
ENA: European Nucleotide Archive
FABI: Forestry and Agricultural Biotechnology Institute
FFSC: *F. fujikuroi* species complex
GenBank: NIH genetic sequence database
GeneMark: Gene prediction software
ITS Internal: Transcribed Spacer
LoRDEC: Hybrid error correction program
MAFFT: Multiple Alignment using Fast Fourier Transform
MEGA: Molecular Evolutionary Genetics Analysis
NCBI: National Center for Biotechnology Information
NRF: National Research Foundation (NRF)
ORF(s): Open reading frame(s)
PhyML: Phylogeny software based on maximum-likelihood principle
PREM: South African National Collection of Fungi
RNA: Ribose Nucleic Acid
rRNA: Ribosomal Ribo Nucleic Acid
SARChI: South African Research Chairs Initiative
SMS: Smart Model Selection in PhyML
SNAP: Web-based tool for identification and annotation of proxy Single Nucleotide Polymorphisms
SPAdes: St. Petersburg genome assembler
SSPACE: SSAKE-based Scaffolding of Pre-Assembled Contigs after Extension program
tRNA: Ribo Nucleic Acid
USA: United States of America
